# Spermatozoan Metabolism as a Non-Traditional Model for the Study of Huntington’s Disease

**DOI:** 10.3390/ijms23137163

**Published:** 2022-06-28

**Authors:** Meghan Lawlor, Michal Zigo, Karl Kerns, In Ki Cho, Charles A. Easley IV, Peter Sutovsky

**Affiliations:** 1 Division of Animal Science, University of Missouri, Columbia, MO 65211, USA; mmlk5g@mail.missouri.edu (M.L.); zigom@missouri.edu (M.Z.); kkerns@iastate.edu (K.K.); 2 Department of Animal Science, Iowa State University, Ames, IA 50011, USA; 3 Department of Environmental Health Science, College of Public Health, University of Georgia, Athens, GA 30602, USA; inkicho@outlook.com (I.K.C.); cae25@uga.edu (C.A.E.IV); 4 Regenerative Bioscience Center, University of Georgia, Athens, GA 30602, USA; 5 Department of Obstetrics, Gynecology and Women’s Health, University of Missouri, Columbia, MO 65211, USA

**Keywords:** Huntington’s disease pathway, male infertility, spermatozoa, zinc-containing/interacting proteins, Huntington’s disease model

## Abstract

Huntington’s Disease (HD) is a fatal autosomal dominant neurodegenerative disease manifested through motor dysfunction and cognitive deficits. Decreased fertility is also observed in HD animal models and HD male patients, due to altered spermatogenesis and sperm function, thus resulting in reduced fertilization potential. Although some pharmaceuticals are currently utilized to mitigate HD symptoms, an effective treatment that remedies the pathogenesis of the disease is yet to be approved by the FDA. Identification of genes and relevant diagnostic biomarkers and therapeutic target pathways including glycolysis and mitochondrial complex-I-dependent respiration may be advantageous for early diagnosis, management, and treatment of the disease. This review addresses the HD pathway in neuronal and sperm metabolism, including relevant gene and protein expression in both neurons and spermatozoa, indicated in the pathogenesis of HD. Furthermore, zinc-containing and zinc-interacting proteins regulate and/or are regulated by zinc ion homeostasis in both neurons and spermatozoa. Therefore, this review also aims to explore the comparative role of zinc in both neuronal and sperm function. Ongoing studies aim to characterize the products of genes implicated in HD pathogenesis that are expressed in both neurons and spermatozoa to facilitate studies of future treatment avenues in HD and HD-related male infertility. The emerging link between zinc homeostasis and the HD pathway could lead to new treatments and diagnostic methods linking genetic sperm defects with somatic comorbidities.

## 1. Introduction

Huntington’s disease (HD) is an autosomal dominant, late-onset neurodegenerative disease caused by CAG repeat expansion in exon 1 of the Huntingtin gene on chromosome 4. It is characterized by disordered movement and cognitive decline in both humans and animal models [1]. The worldwide prevalence of HD in the population of European descent is ~12 per 100,000 individuals [2] and is less common in African, Chinese, and Japanese descent [3]. The age of onset of the motor symptoms can occur from childhood to old age, with the mean age of onset ~45 years [4]. A CAG repeat of 36 or more is pathogenic, with 36–39 repeats conferring reduced penetrance, while 40 and more repeats include complete penetrance with full clinical expression [5,6]. A premotor onset of HD has been suggested by recent evidence [5]. Wild-type Huntingtin (HTT) is a large (350 kDa), ubiquitously expressed protein made up of polyQ N-terminus (as the result of CAG repeats) and several consensus areas called HEAT repeats [7]. HTT is highly expressed in striatal neurons of the central nervous system (CNS), shuttling between cytoplasm and nucleus [8,9,10]. Known functions of HTT are: (i) early embryonic development [11,12] and neurogenesis [13]; (ii) protein scaffolding [14], intracellular trafficking [15], and controlling spindle orientation in neuronal cells [16]; (iii) transcriptional regulator [17,18]; (iv) it is required for proper function of cortical and striatal excitatory synapses [19], and (v) autophagy [20]. A more descriptive overview of HTT functions can be found in [21]. Mutant Huntingtin (mHTT), caused by a minimum of 36 CAG trinucleotide repeats in the Huntingtin gene, results in a loss of efferent medium spiny neurons (MSNs) due to degeneration of striata [22]. The elongated polyglutamine tract at the amino terminus of mHTT affects posttranslational modification of HTT, resulting in altered subcellular distribution, stability, cleavage, and its function [23]. The presence of mHTT disrupts transcription in the brain as well as in peripheral tissues such as muscles and blood [24].

To date, there are numerous cellular (in vitro), invertebrate, and small animal models to study HD including human cellular models [25], *C. elegans* [26], *D. melanogaster* [27], and widely used mouse models [28]. Despite providing valuable knowledge on HD pathology, these models do not always faithfully recapitulate human disease pathology and/or adequately predict clinical response to treatment. Large animal models of HD, spanning the gap between small animal models and humans have been developed in the last decade and currently include sheep [29], minipig [30,31], and macaque [32,33]. The strengths and weaknesses of each said large animal model are discussed in [34]. The subject of the present review, i.e., the unexpected link between the HD pathway and male reproductive function, emerged from our ongoing research on sperm capacitation—a complex signaling and cell structure remodeling process that endows spermatozoa traveling within the female oviduct with fertilizing ability. In our continuous efforts to fully understand the process of sperm capacitation and particularly the role of zinc ions in its regulation, we recently performed a comparative proteomic study of zinc-containing/binding proteins (further zincoproteins) between ejaculated and capacitated spermatozoa [35]. To our surprise, we found that the Huntington’s and Parkinson’s disease pathways were the most represented in the sperm zincoproteome, making the spermatozoon a candidate model for studying neurodegenerative diseases.

This review aims to provide a background on HD pathogenesis, explore the comparative role of zinc in both neuronal function and sperm capacitation, and discuss sperm proteins encoded by genes implicated in HD to facilitate future treatment avenues.

## 2. Mechanisms of HD Pathogenesis

**Figure 1:**
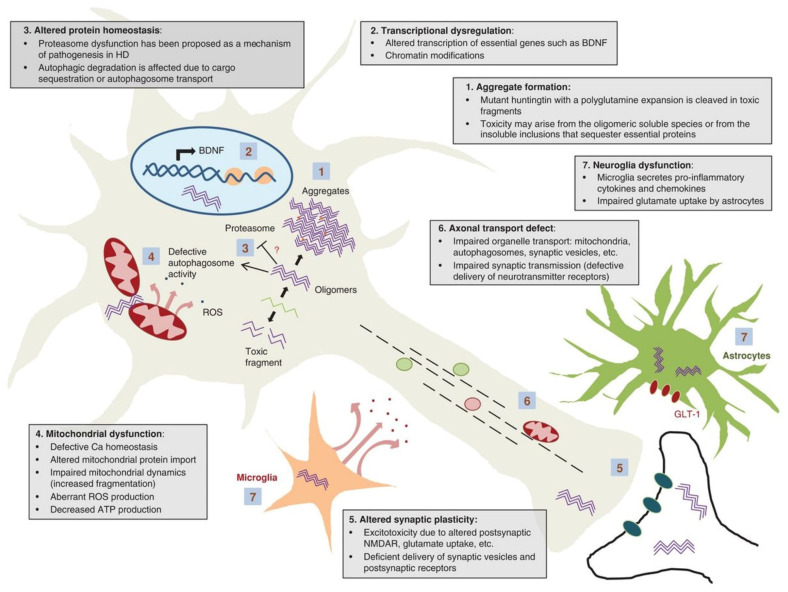
Selected mechanisms of HD Pathogenesis. (**1**) Aggregation of mutant huntingtin, (**2**) Transcriptional dysfunction, (**3**) Proteasomal and autophagy dysfunction, (**4**) Mitochondrial dysfunction, (**5**) Alteration of synaptic plasticity, (**6**) Defective axonal transport, (**7**) Microglial dysfunction. The nuclear transport failure is not depicted. The figure was adapted from Jimenez-Sanchez et al. (2017) [37] and is replicated with permission from Cold Spring Harbor Laboratory Press, obtained on 15 December 2021.

This section aims to provide concise background on HD pathogenesis rather than to give a detailed review, which is provided elsewhere [1,5,6,36,37,38]. Due to the immense complexity of its pathogenesis, implications of HD within striatal neurons are still under investigation; however, there are currently seven proposed mechanisms of pathogenesis, as depicted in Figure 1.

The hallmark of HD is the presence of protein aggregates (1). These are initiated by N-terminal proteolytic cleavage of mHTT into N-terminal polyglutamine fragments that form amyloid fibrils and large inclusions. The cytotoxicity of N-terminal polyglutamine fragments is a question of debate; however, evidence suggests that N-terminal mHTT oligomers are toxic and the toxicity is mitigated by their aggregation into inclusions [39,40]. mHTT aggregates are found in both nuclei and perinuclei (cytoplasm) of a neuron, and it seems that nuclear aggregates are relatively benign when compared to cytoplasmic mHTT aggregates [41].

Binding and inactivation of a number of polyglutamine-containing transcription factors result in altered gene transcription (2), influencing a wide spectrum of cellular functions on the level of transcriptional networks. For instance, mHTT actively transports repressor peptide REST into the nucleus, where it forms a repressor complex on the *BDNF* gene, and reduces levels of BDNF protein—a striatal neuronal pro-survival factor, thus increasing the susceptibility of the striatum to HD [42]. Other transcription-related molecules that mHHT interacts with are: (i) transcription factors: CREB, CBP, SP1, NF-κB, NEUROD1, TP53, PCG-1α; (ii) transcription activators/repressors: TAFII130, CA150, NCOR, REST/NRSF; and (iii) nuclear receptors: LXRα, PPARγ, TRα1 [36]. MHHT interferes with gene expression on the epigenetic level by inducing an imbalance between histone acetylation and deacetylation, DNA methylation and demethylation, and non-coding RNA levels [43].

Another consequence of polyglutamine-expanded mHTT expression is compromised protein degradation systems (3). Impairment of ubiquitin-proteasome system (UPS) is explained by sequestration of UPS components into inclusions or interaction between (and saturation of) proteasomes and aggregation-resistant mHTT [44]. The impaired proteasomal activity is initially compensated by the lysosomal autophagy system that eventually becomes compromised with time in chronic mHTT expression [44,45]. The impairment of mHTT recognition by the cell, endoplasmic reticulum dysfunction in its interaction with N-terminal polyglutamine fragments, as well as disturbance of autophagy regulation by mHTT, ultimately leads to a systemic failure of proteostasis in neurons [46].

Mitochondrial dysfunction (4) is illustrated through decreased transcription of mitochondrial genes due to repression of PGC-1α (peroxisome proliferator-activating receptor coactivator-1α), which regulates the expression of genes mediating mitochondrial biogenesis and respiration, leading to increased susceptibility to oxidative stress and neuronal degeneration [47,48]. Furthermore, mHTT induces an imbalance in mitochondrial dynamics by increasing the transcription of DRP1, a fission-mediating GTPase, resulting in the accumulation of the impaired mitochondria in the cytoplasm [49,50]. In addition, mHTT interacts with both the outer and inner mitochondrial membrane, resulting in defective Ca^2+^ regulation and buffering capacity, and altering the import of mitochondrial proteins due to inhibition of TIM23, respectively [5,37]. The results of such interactions are failures in complex II and IV of the respiratory chain; an influx of Ca^2+^ into mitochondria, promoting the release of cytochrome C; increased ROS production; and ATP synthesis defect [47]. On the macromolecular level, mHTT inhibits mitophagy by affecting the formation of mitophagy initiation protein complexes [51] and impedes axonal transport of mitochondria [52].

Alteration of synaptic plasticity (5) is likely due to mHTT aggregates blocking axons, sequestration of motor proteins, or wild-type HTT loss of function. Mutant Huntingtin impedes the delivery of GABA and AMPA receptors to neuronal membranes, therefore inhibiting synaptic excitability, as well as transport and release of BNDF or retrograde transport of its receptor TrkB, necessary to promote survival signals in the neuronal cell body [50,53]. Excitotoxicity is a detrimental consequence of altered NMDAR signaling and glutamate uptake due to mHTT expression [50,53]. Excitotoxicity is instigated through excess extracellular glutamate via increased glutamate release or impaired uptake due to downregulation of GLT1 [37,54]. This leads to constituent stimulation of NMDAR and ultimately the death of striatal neurons. Excitotoxicity is central to other neurological disorders including Alzheimer’s disease, Parkinson’s disease, and amyotrophic lateral sclerosis (ALS/Lou Gehrig’s disease) [54].

Fast axonal transport of organelles (6) including mitochondria, autophagosomes, and synaptic vesicles is disrupted by mHTT. Impaired transport of organelles occurs either by the direct interference of mHTT with motor protein function of dynein, kinesin, and actin; and/or indirect interaction of mHTT with Huntingtin-Associated Protein 1 (HAP1) through the disintegration of the HAP1/DCTN1 complex followed by its detachment from the microtubules resulting in suppression of the rapid axonal transport [36].

Microglial and astrocyte function (7) is dysregulated by mHTT by inducing the release of proinflammatory cytokine IL-6 [55]. As mentioned earlier, mHTT positively regulates the NF-κB signaling pathway resulting in the release of pro-inflammatory cytokines and chemokines such as IL-6 and IL-8, respectively [56]. Cytokine profile of HD patients had increased levels of IL-4, IL-10, and TNF-α with the disease progression when compared to the control group [57]. Immune and inflammatory changes also occur in peripheral organs during HD development and progression [58].

Last but not least, mHTT disrupts the nuclear transport cycle by sequestering RanGAP1, NUP62, and NUP88 in mHTT aggregates [59].

## 3. Treatments of HD

The same as in the previous section, the purpose of this section is to give a concise overview of currently available treatments, with an in-depth review available elsewhere [1,6,37,60,61]. Current pharmaceutical treatments approved by the FDA in the United States remain symptomatic, only targeting motor, cognitive, and psychiatric symptoms. No disease-modifying treatments are currently available [1].

### 3.1. Symptomatic Treatment Approaches

Symptomatic treatment management aims to improve HD patients’ quality of life. Two examples include Tetrabenazine and Deutetrabenazine, which aid in the management of chorea symptoms caused by neurodegeneration in HD. Chorea, which occurs in early-onset and worsens over time, is characterized by jerky, involuntary movements caused by generalized muscle contraction. Active ingredients in Tetrabenazine and Deutetrabenazine decrease uptake of monoamines (dopamine, serotonin, norepinephrine) in synaptic vesicles by binding and inhibiting Vesicular Monoamine Transporter type-2 (VMAT2) receptors, leading to depletion of monoamines from nerve terminals [62,63]. Tetrabenazine is often characterized by potential adverse effects including hypotension, hyperprolactinemia, suicidal thoughts, and depression [63]. Deutetrabenazine, a derivation of Tetrabenazine, is notably safer than Tetrabenazine as indicated by the mitigated prevalence of adverse events including depression, somnolence, and insomnia [62]. In addition, it is comparably effective at lower dosages.

Cognitive symptoms including decreased processing speed, poor attention, poor problem-solving abilities, and poor memory retrieval can manifest decades before the onset of motor symptoms in HD patients. To date, there is no effective therapy for HD-associated dementia. Metamatine that is used to treat Alzheimer’s dementia seems promising with a potential neuroprotective effect with long-term use [64].

The most commonly reported psychiatric symptom in HD patients is major depressive disorder [65]. Although there is no guide to pharmacotherapy of HD depression, selective serotonin reuptake inhibitors have been shown to be beneficial by HD patients [66].

### 3.2. Clinical Trials

As stated previously, no treatment for HD is currently available that would target the disease itself. However, the potential efficacy of numerous treatment approaches for HD is currently being tested through clinical trials. These are also available at ClinicalTrials.gov (accessed on 16 March 2021). A summary of selected interventions and their mechanisms are discussed below.

#### 3.2.1. Genetic Manipulation

Various approaches, as listed in Table 1, aim to destroy or inactivate HTT/mHTT translation through gene silencing and are currently undergoing clinical trials. Both allele-specific and non-specific approaches have proven effective in mouse models. Allele-specific and non-specific mouse models show a significant correlation between reduced mHTT expression and decreased atrophy, improved motor function, and improved cognition [61]. Although such methods prove effective, drug developers may ultimately need to combine gene silencing with immunomodulatory drugs to complement immune activation. The anti-semaphorin 4D antibody VX15/2503 is a monoclonal antibody against semaphorin 4D, a transmembrane protein modulating microglial activation, oligodendrocyte viability, and permeability of the blood/brain barrier [67].

Allele-specific, WVE-120101, and WVE-120102 are mHTT-specific lowering antisense drugs that target SNP variants linked to CAG expansions, leading to pre-mRNA degradation [61,67]. mHTT-specific drugs by Imperial College London and Sangamo Therapeutics under preclinical trials integrate zinc-finger domains into adeno-associated viruses (AAVs) and lentiviral vectors to modify effector elements and repress transcription of mHTT. In mouse models, a 78% reduction of mRNA was achieved, resulting in reduced mHTT aggregate levels if administered early in disease onset [61].

Allele-non-specific, HTT-lowering miRNA-based gene silencing approaches include AMT-130 and VY-HTT01. Both harness a particular RNAi pathway to silence both HTT and mHTT expression through the use of viral vectors [61,67]. RNA-induced silencing complexes target mRNA for degradation, yielding reduced protein expression [61]. After an exact dose of AMT-130 to achieve knockout was identified, the FDA granted fast-track designation to the first AAV gene therapy approved for human trials. In monkey models, VT-HTT01 has shown 50% improvement in motor function [61]. Direct injection of AMT-130 and VY-HTT01 AAVs or lentiviral vectors could provide lifelong silencing of mHTT [61,67]. An additional non-specific drug, RG6042, aids in the prevention of toxic fragment buildup, as it silences the expression of all HTT forms to inhibit the synthesis of toxic byproducts and prevents neurodegeneration [67]. This method has proven effective through the significant correlation between lowered mHTT levels and function, cognitive, and motor scores [61]. For a more in-depth review of mHTT lowering therapies, see [68].

#### 3.2.2. Mitochondrial Function and Biogenesis

Three clinical trials are pending with relevance to increase mitochondrial function: (i) Fenofibrate is a peroxisome proliferator-activated receptor (PPAR) agonist that may induce activation of PGC-1α and aid in mitochondrial biogenesis [69]; (ii) Triheptanoin is a C7 fatty acid oil that is hydrolyzed into acetyl-CoA and propionyl-CoA and may provide substrates to the Krebs cycle, thus increasing ATP availability and correcting the bioenergetic profile in the HD brain [70]; and (iii) Metforminis an AMP-activated protein kinase (AMPK) activator commonly used to treat type 2 diabetes that has been shown to be protective of polyQ-expressing models [71,72], increasing the lifespan of HD transgenic mice [73] and increasing cognitive function in HD patients [74].

#### 3.2.3. Modulation of BDNF Levels

A clinical trial of Pridopidine, a first-in-class sigma-1 receptor (S1R) agonist and dopamine receptor antagonist [75], is underway. S1R plays a key role in neuroprotection through the increased production of BDNF, a striatal neuronal pro-survival factor, levels.

#### 3.2.4. Synaptic Modulation

A clinical trial of Neflamapimod, a small molecule that can penetrate the brain and inhibit the enzyme P38α (MAPK14), is underway. P38α is typically involved in regulating inflammation, and its chronic activation negatively affects nerve cell communication due to excessive inflammation [76].

#### 3.2.5. Stem Cell Therapies

Considerable potential for the treatment of neurodegenerative diseases is represented by stem cell therapies with neuronal progenitor cells derived from induced pluripotent stem cells (iPST) [77]. Studies have shown that transplanted iPST can differentiate into functional neurons and improve partial striatal function and metabolism after implantation in a rat HD model [78,79]. Three clinical trials are pending to assess the dose, safety, and efficacy of intravenous delivery of stem cells in human HD patients [1].

## 4. Zinc Homeostasis, Reproduction and Huntington’s Disease

Both the brain and the testes contain high zinc ion (Zn^2+^) concentrations: brain ~70 µg·g^−1^ dry weight [80] and testes ~74.6 µg·g^−1^ dry weight [81]. An increased concentration leads to neurotoxicity [82]/reprotoxicity [83], while decreased concentration leads to impaired brain functioning [84] and impaired spermatogenesis [85], respectively. Therefore, zinc homeostasis needs to be tightly regulated in order for both the central nervous system and reproduction to function properly. In this section, similarities in neuronal and sperm zinc homeostasis will be discussed.

### 4.1. Neuronal Zinc Homeostasis

**Figure 2:**
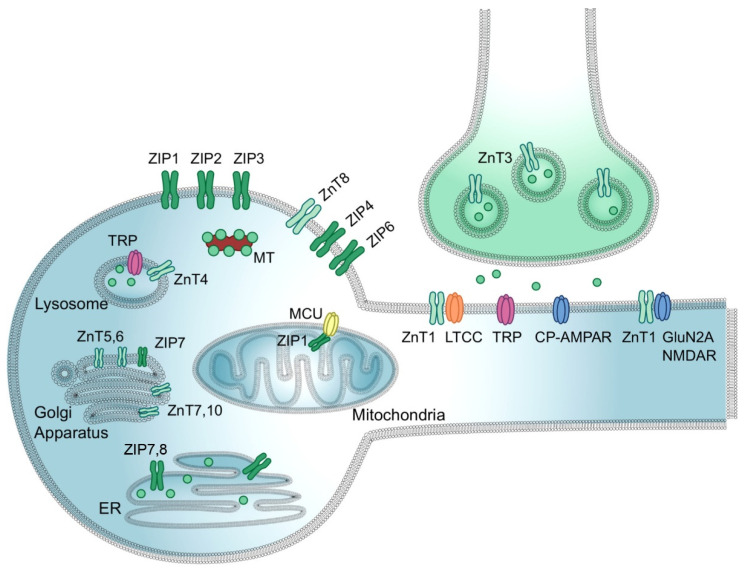
Zinc transport in neurons. Zn^2+^ is loaded into presynaptic vesicles via ZnT3, released into the synaptic cleft, and conducted to the cytosol of a postsynaptic neuron via L-type calcium channels (LTCC), transient receptor potential channels (TRP), NMDA receptors, and calcium-permeable AMPA receptor (CP-AMPAR). Within the neuron, Zn^2+^ uptake to/release from specific organelles is mediated by organelle-specific ZIPs and ZnTs. Zn^2+^ import into mitochondrial matrix is mediated by mitochondrial Ca^2+^/cation uniporter (MCU) coupled with ZIP1. Free Zn^2+^ concertation within the cytosol is buffered by metallothioneins (MT). The figure was adapted from Krall et al. [86] and replicated with permission from Elsevier Ltd., license #5203200109216, obtained on 6 December 2021.

Zinc is one of the most prevalent and essential elements involved in brain function, tightly regulated for its proper function, and a potent neurotoxin when dysregulated. Zinc plays several roles in brain function including neurotransmission and sensory processing, and activation of both pro-survival and pro-death neuronal signaling pathways [86]. Zinc homeostasis in the brain is maintained by three groups of proteins: (i) zinc importers (ZIPs), transporting Zn^2+^ into the cytosol; (ii) zinc transporters (ZnT), transporting Zn^2+^ out of cytosol into both membranous organelles and to the extracellular environment; and (iii) metallothioneins (MTs), actively increasing/decreasing concentration of free/unbound Zn^2+^ in the cytosol (Figure 2) [87,88]. Expression of zinc transporter 3 (ZnT3) localized on the membrane of synaptic vesicles is required for the transport of zinc from the cytosol into presynaptic vesicles. The concentration of vesicular zinc (10–15% of total brain zinc) is dependent on the abundance of ZnT3 transporters [89]. In excitotoxic settings and neurodegeneration, MTs metal-binding redox-sensitive proteins mobilize large amounts of Zn^2+^ to re-establish vesicular neuronal zinc homeostasis [90]. PrPc (cellular prion protein) promotes zinc uptake as PrPc binds the ion via octapeptide repeats to facilitate the transport of the metal across the membrane through the AMPA receptor channel [91]. PrPc contributes to neuronal zinc homeostasis in three ways: by acting as a zinc sink, prompting zinc uptake via endocytosis into the postsynaptic neuron, and serving as a zinc sensor, monitoring levels of the ion in the synaptic cleft [91]. If extracellular zinc concentration rises above a certain threshold, a signaling cascade is triggered to increase the transcription of metal transporter genes. Excess zinc must be cleared from the synaptic cleft to prevent neuronal damage [91]. In this way, PrPc acts as a zinc sensor in coordination with AMPA receptors to maintain healthy levels of zinc in the synaptic cleft.

Vesicular zinc serves as a neuromodulator in glutamatergic neurons for glutamate signaling and cognitive ability [89]. In its role as a neuromodulator, Zn^2+^ is released during synaptic transmission and binds to pre- or postsynaptic membranes, essentially translocating from presynaptic terminals to postsynaptic neurons (Figure 2) [92]. When released into the synaptic cleft with glutamate during excitatory neurotransmission, Zn^2+^ exerts downstream inhibitory effects on NMDAR and AMPAR [90]. In this way, Zn^2+^ protects neurons from excitotoxicity and attenuates excess pre-synaptic glutamate [89,90].

Zn^2+^ is critical for the activation state of matrix metalloproteinases (MMPs) that are involved in BDNF maturation from its precursor form proBDNF [93]. Pro-BDNF/BDNF balance is critical for neuronal function, as Pro-BDNF activates neuronal death-related pathways and BDNF promotes long-term memory processes and neuronal survival [90]. Zn^2+^ also activates BDNF receptor TrKB activating downstream signal transduction [94].

### 4.2. Zinc Dysregulation in HD

Zinc homeostasis is dysregulated in a wide range of neurological diseases including Alzheimer’s disease (AD), amyotrophic lateral sclerosis (ALS), traumatic brain injury (TBI), hypoglycemia-induced neuronal death, ischemia-induced neuronal death, schizophrenia (SCZ), major depressive disorder, and Parkinson’s disease (PD), as well as HD [90,92,95].

Zn^2+^ dysregulation is believed to contribute to HD in a condition characterized by the occurrence of a chronic glutamatergic overdrive [90]. In such a condition, the aberrant release of glutamate and Zn^2+^ from presynaptic vesicles occurs. Glutamate stimulates NMDAR that causes Ca^2+^ influx to postsynaptic neurons, while Zn^2+^ enters via respective channels (Figure 2). Increased cytosolic Ca^2+^ concentration triggers irreversible cytotoxic effects including mitochondrial dysfunction, oxidative stress, and reduced ZnT3 expression. Following Zn^2+^ uptake via mitochondrial Ca^2+^/cation uniporters (MCU), the function of electron transport chain complexes and α-ketoglutarate dehydrogenase is inhibited, leading to ROS production and metabolic failure. Mitochondrial uptake of Zn^2+^ also triggers activation of mitochondrial permeability transition pores, promoting neuronal apoptosis [90]. Cytosolic Zn^2+^ activates neuronal isoform of nitric oxide synthase (nNOS), promoting increased NO production, as well as NADPH oxidase resulting in overproduction of superoxide anion ($O_{2}^{-}$). Free $O_{2}^{-}$ combined with NO yields peroxynitrite (ONOO^−^), a neurotoxic reactive nitrogen species (RNS) [90]. Zn^2+^-driven ROS and RNS promote further metal release from MTs, resulting in a positive feedback loop of dyshomeostasis. Motor neurons are especially vulnerable to oxidative stress and dysregulation because they express CP-AMPARS, which offers a rationale for the degeneration of motor neurons [90]. Other factors leading to neuronal failure include Zn^2+^-driven NADPH oxidase activation, NAD^+^ depletion, GAPDH inhibition, ATP breakdown, and outward K^+^ currents [90]. As a result, patients often express a mix of neurotoxic proteins including mutant prion proteins, tau, and synuclein [90].

In addition, studies indicate that vesicular zinc deficiency also causes neuronal apoptosis and memory deficit in HD. mHTT inhibits the binding of Sp2 to the ZnT3 promoter, downregulating ZnT3 expression in motor neurons [89]. Transgenic mice models with a loss of synaptic vesicular zinc and disruption of vesicular zinc homeostasis have shown a decrease in dendritic spines, defective presynaptic synaptosome-associated protein 25 (SNAP25) and postsynaptic PSD95, early synaptic dysfunction, neurodegeneration, and cognitive decline in HD [89]. In addition, a loss of vesicular zinc leads to glutamate-mediated excitotoxic neuronal death [89].

### 4.3. Role of Zinc in Sperm Maturation

An increasing level of attention has been given to the role of Zn^2+^ in male reproduction since our last review [96]. Zn^2+^ is essentially necessary for almost every step of sperm maturation. Similarly, as in neuronal cells, zinc trafficking is mediated by ZnTs and ZIPs during sperm maturation. In the human male reproductive organs, ZnTs are expressed in the testis (Sertoli cells, ZnT1) and epididymis (ZnT1,2), but no ZnT expression was reported in spermatozoa [97]. On the contrary, ZIP1, 5, 6, and 8 are expressed in human testicular and epididymal spermatozoa and the epithelial/stromal cells of the testis and epididymis.

Starting with spermatogenesis, the Zn^2+^ requirement has been shown for the maintenance of germ cells and progression of spermatogenesis in the Japanese eel [85], and spermiogenesis activation in *C. elegans* [98,99]. Further, Zn^2+^ is vital for the maintenance of normal spermatogenesis in rats [100,101,102], mice [103], and rams [104]; by maintaining male germ cell proliferation [100,101,102], by expression of testis germ cell-specific genes during the cell differentiation and spermatogenesis [104], and by regulating Leydig cell generation [100]. Zn^2+^ accumulates in spermatocytes with its presence in the nucleus and chromatin [105], where it participates in the histone for protamine substitution event [98]; is required for the proper functioning of zinc finger protein (ZFP) transcription factors, such as ZFP185 or ZFP318 [106,107]; and stabilization of sperm chromatin with zinc bridges [108]. Furthermore, Zn^2+^ stabilizes nascent outer dense fibers (ODF) of the sperm flagellum and protects them from premature oxidation [109,110]. Zinc deficiency has therefore an adverse effect on spermatogenesis and the general abnormalities include hypogonadism and Leydig cell damage resulting in a deficiency of sex hormone production and impaired spermatogenesis, as well as inflammation, antioxidant depletion, and sperm death leading to male infertility [111]. The main mechanism of zinc deficiency seems to be oxidative stress induced by ROS that causes depletion of antioxidants, sperm DNA fragmentation, lipid peroxidation, and apoptosis; and, consequently, poor sperm quality [111].

**Figure 3:**
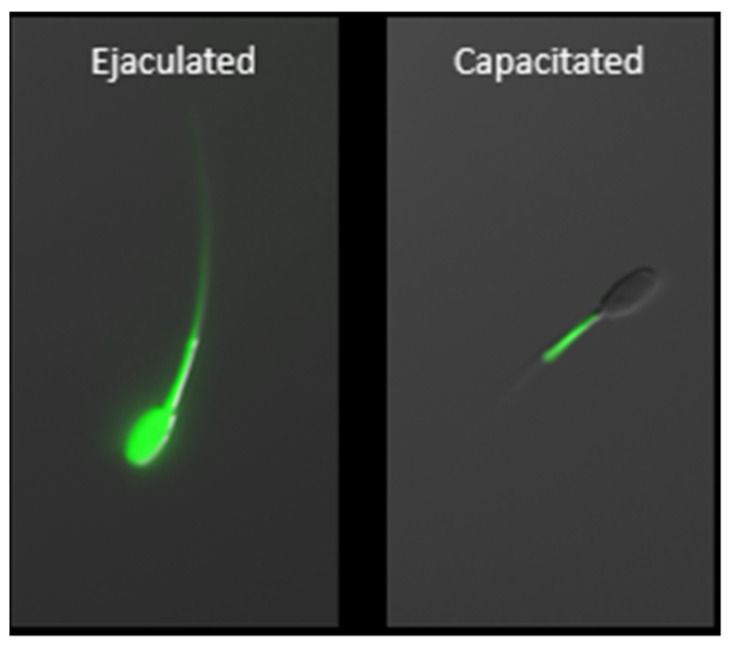
Zinc localization in boar ejaculated and capacitated spermatozoa using fluorescent probe FluoZin^TM^-3, AM. Capacitation-related efflux of zinc ions from the sperm head and principal piece is a prerequisite for achieving capacitated, hyperactivated status. Following capacitation, zinc is localized exclusively within the mitochondrial sheath where it is functional, thus indicative of the importance of zinc homeostasis in the sperm mitochondria. The scale bar represents 10 µm.

During the epididymal transit of rat spermatozoa, approximately 60% of Zn^2+^ content is lost from epididymal spermatozoa [112,113] and reabsorbed by the epididymal epithelium [114]. This maturation step allows for the oxidation of sulfhydryl groups thus stiffening the ODF, and is mandatory to obtain functional ODF, a prerequisite for progressive motility. A thorough study was performed on zinc trafficking in human spermatozoa during the passage through the human male reproductive tract [97]. The authors found that contrary to the aforementioned rat studies, the total human sperm zinc content increases as spermatozoa pass through the male reproductive tract (2.56, 12.58, and 40.48 ng Zn^2+^/10^6^ sperm in testicular, epididymal, and ejaculated spermatozoa, respectively). The autometallography assessment of Zn^2+^ accumulation revealed that Zn^2+^ is preferentially accumulated within the residual cytoplasm of immature testicular and epididymal spermatozoa, while in the ejaculated spermatozoa Zn^2+^ is localized predominantly in the midpiece, connecting piece, and flagellum [97]. Thus, Zn^2+^ trafficking likely plays a role in post-testicular sperm maturation including cytoplasmic rearrangement and chromatin condensation as previously suggested [115]. For the ejaculated human spermatozoa, the same trend was observed by another collective of authors [116] where 93% of the total zinc content was allocated to the flagellum. For the porcine (Figure 3) and bull spermatozoa, we reported that zinc is present throughout the entire spermatozoa; however, we did not perform organelle- or compartment-specific quantification of zinc content [117]. Higher content of flagellar Zn^2+^ has been negatively correlated with motility [97,116] and therefore it is reasonable to believe that Zn^2+^ may regulate sperm motility via a direct or indirect mechanism. We have previously suggested a model for how this may be orchestrated [96] and this model was further developed by another group [118]. Further, incorporation of Zn^2+^ into spermatozoa during ejaculation was proposed to have sperm chromatin stabilization effects in humans [119,120,121] and delay acrosomal exocytosis in humans [122]. It may also maintain plasma membrane structure and function, as shown in a model membrane system as well as in erythrocytes of multiple species [123,124], and protect against free radicals in human and goat spermatozoa [125,126].

During the last stage of sperm maturation, known as capacitation, that occurs in vivo within the oviductal epithelium sperm reservoir [127], Zn^2+^ is gradually released from spermatozoa (Figure 3), causing activation of signal transduction pathways resulting in hyperactivation, acrosomal and sperm surface remodeling, and increased affinity to zona pellucida. As reported previously, Zn^2+^ removal is an absolute necessity for spermatozoa in achieving full fertilization potential [96,97,116,117,118,128]. These zinc localization patterns have been termed the sperm zinc signature, with four patterns discovered initially in boar, bull, and man [117]; with subsequent follow-up, zinc localization to the sperm acrosome in bull [128]. These localization patterns are indicative of key stages of the sperm capacitation process. We have shown that the spermatozoa release from glycans, mimicking the oviductal epithelium reservoir in response to the oocyte-produced chemoattractant progesterone, is inhibited in the presence of biologically relevant millimolar Zn^2+^ concentrations, found in seminal plasma. This is notable because progesterone (P_4_) is contained in oocyte follicular fluid when released at the time of ovulation, thus guiding spermatozoa to the final site of fertilization. Further, we have shown that high, biologically relevant levels of Zn^2+^ directly inhibit the activity of matrix metalloproteinase (MMP) 2 and 9, as well as ACR and 26S proteasome [128]. Proteasome-inhibited bovine spermatozoa fail to undergo acrosomal exocytosis likely due to insufficient acrosomal remodeling [129]. It has been proposed that the inner acrosomal membrane localization of both MMP2 and ACR predestines these enzymes for a role in the ZP degradation at fertilization [130]; however, participation of MMP2 in the acrosomal remodeling also seems to be plausible and would be in support of previous findings [122]. Inhibition of said enzymes by millimolar Zn^2+^ concentration might be an evolutionarily conserved polyspermy defense mechanism, given that the sperm-activated oocytes of many species release billions of zinc ions at the time of fertilization during an event referred to as the zinc spark (Xenopus [131], mice [132,133], cattle [134], and human [133]).

## 5. HD Implications in Male Fertility

Similar membrane characteristics and features are shared between neurons and spermatozoa; many neuronal receptors are also present in spermatozoa which have been labeled as “neurons with tails” [135]. Spermatozoa share excitability functions with neurons, although they lack the synaptic mechanism [136]. Several neural receptors in spermatozoa have been linked with sperm function, such as acrosomal exocytosis mediated by dopamine and 5-HT receptors [137], or motility associated with purinergic [138], nicotinic [136,139], angiotensin II [140], cannabinoid [141], and olfactory receptors [142]. It is, therefore, no surprise that in idiopathic infertile men, asthenozoospermia was associated with polymorphism related to neurotransmission genes such as *HTR2A*, *MAOA*, and *SLC18A1* [143].

The brain and testes share the highest similarity in *HTT* gene expression patterns [144], with both having the highest HTT expression among all body tissues [145]. Similarly, in HD individuals, the brain and testes have a high level of somatic mosaicism of CAG repeats in *HTT*. The CAG repeat track was shown to progressively increase with the age in sperm and lymphocytes of human patients, HD mice, and HD monkeys [146,147,148,149,150]. During spermatogenesis, HTT is localized in the cytoplasm of spermatogonia, while nuclear localization was observed in spermatids and spermatozoa [151]. Nuclear delocalization of HTT during meiosis suggests its role in it. Indeed, male transgenic mice expressing solely mHTT were characterized by infertility, testicular atrophy, aspermia, and massive apoptotic cell death in testes [152]. This was later confirmed in a conditional KO mouse with germ-line specific ablation of *HTT* which resulted in male infertility, with spermiogenesis arrested at step 3 of the Golgi phase and a significant testis protein profile alteration [153]. In humans, specific testicular pathology with reduced numbers of germ cells and abnormal seminiferous tubule pathology were reported in HD patients [154].

Some aspects of male reproduction were examined in the transgenic HD (TgHD) minipig model [30]. The TgHD boars gradually develop the neurodegenerative phenotype accompanied by testicular degeneration within 24 months of age [155]. After reaching one year of age, the TgHD boars had reduced fertility, fewer spermatozoa per unit in the ejaculate, and a significant decline in the number of WT oocytes penetrated by TgHD spermatozoa in vitro [30]. A follow-up report [156] confirmed previous mouse and human studies by showing that TgHD minipigs had testicular tubule atrophy as a result of mHTT accumulation. Multinucleated spermatogenic cells were frequently present and spermatogonia were shrunk with dilated ER, swollen mitochondria, and condensed chromatin, while spermatocytes and spermatids were rarely present. Further, some tubules contained Sertoli cells only, which were characterized by increased density and vacuolization of cytoplasm, dilation of ER, swollen mitochondria, and alterations of nuclear structure [156]. Sperm defects of TgHD minipig included deformation of the mitochondrial sheath, and folded or coiled flagella containing double or triple axoneme with observations of a fused mitochondrial sheet, while mHTT was detected along the whole sperm tail of TgHD boars [156]. In their later report [157], the same group explored whether the HD pathology was connected with a defect in mitochondrial metabolism. They incubated TgHD spermatozoa with different ^14^C substrates of the mitochondrial energy-generating system and monitored the production of ^14^CO_2_. The production of ^14^CO_2_ was significantly reduced in four incubations when compared to WT spermatozoa, implying a non-specific pathology in mitochondrial complex I of the oxidative phosphorylation system and/or in substrate-level phosphorylation of glycolysis, possibly linked with mHTT toxicity [157]. This finding has implications for HD progression monitoring and the development of an efficient strategy for targeted therapy.

### 5.1. Dysregulation of Neurodegenerative Pathway Genes Linked with Subfertility

In a study utilizing a buffalo model, transcriptomic profiling was conducted in low-fertile buffaloes and compared to high-fertile buffaloes [158]. The authors found 709 significantly dysregulated genes in total between low- and high-fertile buffaloes. Of the top 10 downregulated pathways, (i) AD pathway with 28 downregulated genes was the top one, (ii) HD pathway with 25 downregulated genes was the fifth (Table 2), and (iii) Parkinson’s disease with 22 downregulated genes was the eighth-most downregulated pathway. Even though the implications of genes of neurodegenerative pathways in sperm fertility are still unknown, these results offer an interesting avenue for a role exploration of the neurodegenerative pathway genes in sperm physiology.

The same group performed transcriptomic profiling of low-fertile bulls and compared their transcriptome profile to high-fertile bulls [159]. Surprisingly, or not so much at this point, Huntington’s and Parkinson’s disease pathway transcripts were uniquely present in high-fertile bulls, further supporting the hypothesis of the role of neurodegenerative pathways in male fertility.

### 5.2. Zinc-Interacting Proteins of Boar Spermatozoa and Neurodegenerative Pathways

Identification of zinc efflux during mammalian sperm capacitation [117] stimulated further research into characterizing sperm zinc-interacting/zinc-binding proteins (zincoproteins/zincoproteome). In our newest study [35], we identified 1752 zincoproteins with 102 changing significantly during sperm capacitation in vitro. After performing pathway analysis on identified zincoproteins, we made an observation that HD and PD pathways were the two most represented and AD was the tenth-most represented one. HD pathway represented forty-two zincoproteins (Table 3), of which five showed significantly different expression in boar spermatozoa following capacitation (DNAH7, DNAH8, DNAI2, HIP1, and TUBB4B). Their primary critical functions in spermatozoa include ATPase activity, microtubule motor activity, actin filament binding, and cytoskeleton organization. Spermatozoa and sperm capacitation, especially with these five zincoproteins, could be used as a model to explore the effects of HD on spermatozoan zinc homeostasis throughout disease progression.

## 6. Spermatozoon as a Potential Model for Studying HD

The spectrum of similarities between neurons and spermatozoa suggests a potential for developing a very easily manageable alternative cellular model system to study and diagnose neurological disorders such as HD, PD, and AD. Further, spermatozoa can be obtained in a non-invasive manner giving a possibility to screen and monitor the disease progression and/or regression during treatment. Studying these pathways in the male pig, which provides an abundant quantity of spermatozoa necessary for various study techniques, could be advantageous [160], especially since the pig is three times more genetically similar to the human than rodent models on the nucleotide level [161]. Likewise, given the non-invasive nature of semen collection, human spermatozoa could be used to study these neurological disorders as a model directly from a human. Studies on higher-order mammals such as the pig would undoubtedly identify pathways more similar to corresponding human pathways given the current number one model organism for HD is Drosophila [162].

### In Vitro Spermatogenesis Model for Studying HD

Due to limited availability and ethical concerns, studying mechanisms involved in the pathogenesis of HD in human spermatogenesis has been challenging. However, recent development in the stem cell research field, especially induced pluripotent stem cell (iPSC) and directed differentiation helped to derive many tissue-specific cell types that can be used in research. Research on in vitro gametogenesis (IVG) is an active field of research, and the goal is to achieve complete oogenesis and spermatogenesis in culture. A complete in vitro spermatogenesis from pluripotent stem cells resulting in offspring has been reported in the mouse [163,164]. Recently, the first development of blastocyst was reported in non-human primates (NHP) followed by fertilization with in vitro derived spermatid from embryonic stem cells utilizing the same directed-differentiation protocol used in the human [164,165]. The differentiation protocol is being improved [166,167]. The successful generation of fertile male gametes requires a seamless orchestration between male germ cells and supporting cells including Sertoli, Leydig, and peritubular cells where spermatogenesis occurs in a highly organized tubular microenvironment, the seminiferous tubules, which constitute the mammalian testis. Besides generating haploid male gametes, the production of androgens through steroidogenesis is also an important function of the mammalian testis that regulates spermatogenesis as well as secondary male features.

**Figure 4:**
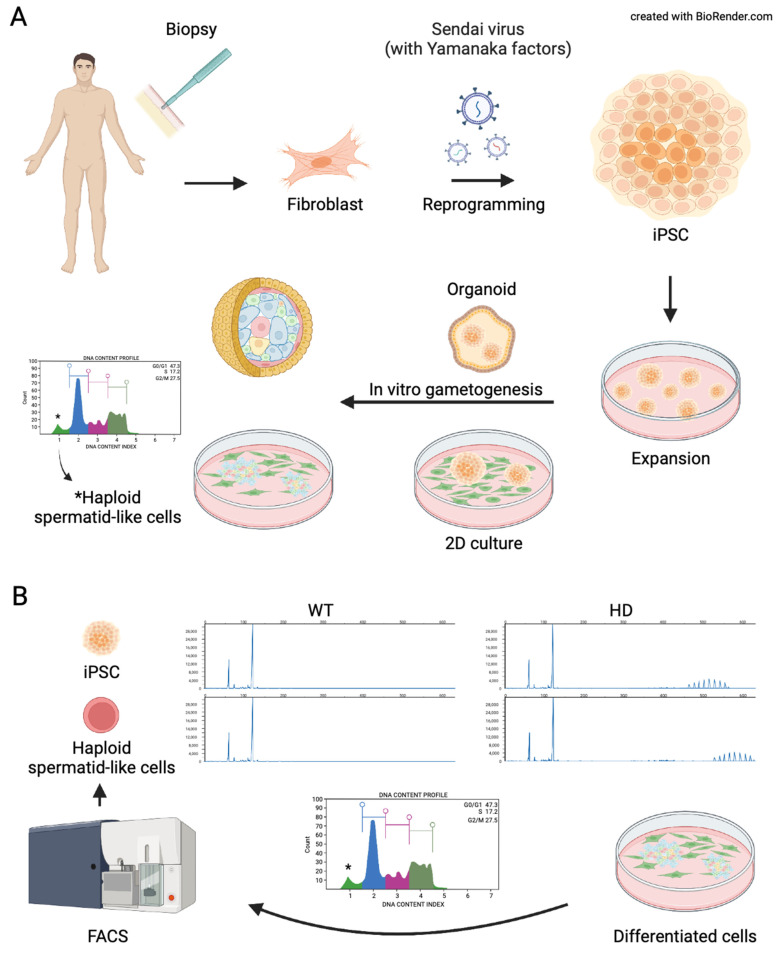
Schematic representation of current models of in vitro spermatogenesis. (**A**) somatic cells can be collected from a patient where they can be reprogramed with reprogramming factors to derive induced pluripotent stem cells (iPSCs). The iPSCs can be preserved, expanded, and induced to produce advanced spermatogenic cells via 2D culture methods or 3D culture methods (3D). (**B**) A schematic representation of a recent study where differentiated spermatogenic cells were used to study developmental mechanisms involved in the pathogenesis of Huntington’s disease. BioRender.com, access on 20 April 2022.

The research on human in vitro spermatogenesis started in the 1960s from the organ culture [168]. In mice, reconstituting embryonic testicular cells has been used to derive and propagate spermatogonial stem cells from embryonic stem cells [169]. In vitro differentiation showed protracted and less efficient differentiation, and derived cells did not enter meiosis even in the prolonged culture [169]. Studies were conducted where both NHP and human pluripotent stem cells (hPSCs) were differentiated into primordial germ cell-like cells [170,171,172,173,174,175,176,177,178,179]. However, most of those studies failed to generate advanced spermatogenic past meiosis cells such as spermatid or spermatozoa. In later studies, hPSCs were differentiated into advanced spermatogenic cells and even haploid cells [164,174,175,180]. Even though haploid spermatid-like cells were derived from hPSCs, elongations have not been reported from 2D culture methods, and due to the ethical problems, fertilizations with in vitro-derived spermatid-like haploid cells have not been reported. However, in a recent study, elongation of spermatid-like cells, fertilization, and development of blastocyst with in vitro-derived spermatid-like cells have been reported in NHP [165]. Up-to-date, fully matured spermatozoa have only been reported by grafting primordial germ cell-like cells (PGCLCs) or spermatogonial stem cell-like cells (SSCLCs) in the testes or in ex vivo organ culture in rodents, NHPs, and humans. Self-organization of the testicular organoid was reported during extended culture with testicular cells that cannot be achieved in 2D culture conditions [181]. Most importantly, meiotic and post-meiotic germ cells were derived from undifferentiated spermatogonia isolated from prepubertal rhesus monkeys in a 3D soft agar culture [182]. A recent report on human 3D testicular organoids with the production of testosterone without the stimulation of hCG and the expression of protamine 1 (PRM1) and Acrosin in male germ cells suggests functional testicular organoids can be reconstructed in vitro by using testicular cells and extracellular matrix (ECM) [181,183]. The recent advancement of in vitro spermatogenesis and different schemes are summarized in Figure 4A.

Besides utilizations in regenerative reproductive medicine wherein in vitro derived advanced spermatogenic cells are used to fertilize oocytes or repopulate testes, a recent study utilized in vitro spermatogenesis to investigate the underlying mechanism of pathogenesis in HD. Trinucleotide repeat (TNR) expansion predominantly occurs through male lineage in HD. Unlike in rodents, where TNR expansion occurs in the post-meiotic stage [184,185], in humans, TNR expansion occurs in pre-meiotic and post-meiotic spermatogenic cells [186]. Those differences could be due to kinetic, biological, life span, and developmental process differences between rodents and humans [187,188]. Because NHP resembles biological and developmental processes during spermatogenesis, a recent study utilized in vitro spermatogenesis to study CAG repeat behavior in spermatid-like cells derived from NHP ESC [189]. The study showed the in vitro model resembles CAG repeat expansion observed in the sperm cells where repeat size is the main factor affecting the CAG repeat instability (Figure 4B) [189].

## 7. Conclusions

The goal of this review was to demonstrate the extensive parallels between neurons and spermatozoa. As “neurons with tails”, spermatozoa represent a unique, high-potential model for the study, diagnosis, and therapy/drug development targeting Huntington’s disease. Future studies should investigate the roles of neurodegenerative disease pathways in mammalian reproduction and how these are involved in regulating and sustaining male fertility. The feasibility and efficacy of this unique model need to be explored as well. In addition, targeting zinc dysregulation and exploitation of zincoproteome involved in spermatozoan capacitation will serve as an avenue for the exploration of potential therapeutic remedies to combat HD. Targeting Zn^2+^ dysregulation might serve as a potential therapeutic remedy to prevent the onset of disease, as altered neuronal zinc homeostasis and spermatozoan metabolism occur in the early stages of HD. Future clinical studies must evaluate the most efficient way of preserving zinc homeostasis and evaluate when, where, and how much Zn^2+^ is ideal for proper neural and reproductive function. In addition, quantification of mitochondrial metabolic biomarkers in spermatozoa, including those linked with HD pathology, could be useful for the diagnosis of early-onset HD. Moreover, in vitro spermatogenesis models can be used to study pathogenesis mechanisms involved during spermatogenesis in HD. Spermatozoa may be suitable noninvasive sampling material for monitoring HD disease progression and therapy. In addition, spermatozoa could be used to monitor the efficacy of preclinical therapeutic treatments.

## Figures and Tables

**Table 1:** Huntingtin lowering therapies targeting DNA and RNA.

Drug/Sponsor	Class	Specificity	Target	Mechanism of Action	Trials
WVE-120101, WVE-120102/WAVE Life Sciences	ASO	Allele-specific	RNA	mHTT-lowering antisense Pre-mRNA degradation	Phase I/II
Imperial College London	ZFP	Allele-specific	DNA	Transcriptional repression	Pre-clinical
Sangamo Therapeutics/Takeda	ZFP	Allele-specific	DNA	Transcriptional repression	Pre-clinical
RG6042 (IONIS-HTTRx)/Roche/Ionis Pharmaceuticals	ASO	Non-specific	RNA	HTT-lowering antisense Pre-mRNA degradation	Phase III
AMT-130/uniQure	RNAi	Non-specific	RNA	HTT-lowering miRNA-based silencing mRNA degradation	Phase I/II
VY-HTT01/Voyager Therapeutics	RNAi	Non-specific	RNA	HTT-lowering miRNA-based silencing mRNA degradation	Pre-clinical

ASO, antisense oligonucleotide; RNAi, RNA interference; ZFP, Zinc Finger protein.

**Table 2:** HD pathway genes, significantly downregulated in low-fertile buffaloes as reported by Paul et al. [158]. Function(s) of the proteins were taken from the UniProtKB database (https://www.uniprot.org/), accessed on 3 May 2021.

Gene	Protein	Function(s)
ATP5A1	ATP synthase subunit alpha, mitochondrial	ADP binding; angiostatin binding; ATP binding; MHC class I protein binding; proton-transporting ATP synthase activity, rotational mechanism; RNA binding
ATP5G1	ATP synthase F(0) complex subunit C1, mitochondrial	lipid binding; proton-transporting ATP synthase activity, rotational mechanism
ATP5G3	ATP synthase F(0) complex subunit C3, mitochondrial	lipid binding; proton-transporting ATP synthase activity, rotational mechanism
ATP5H	ATP synthase subunit d, mitochondrial	protein-containing complex; proton transmembrane transporter activity
BDNF	Brain-derived neurotrophic factor	growth factor activity; nerve growth factor receptor binding; activation of phospholipase C activity; axon guidance; brain-derived neurotrophic factor receptor signaling pathway; collateral sprouting; modulation of chemical synaptic transmission; negative regulation of apoptotic signaling pathway; negative regulation of myotube differentiation; negative regulation of neuron apoptotic process; nerve development; nerve growth factor signaling pathway; nervous system development; neuron projection morphogenesis; neurotrophin TRK receptor signaling pathway; peripheral nervous system development; positive regulation of brain-derived neurotrophic factor receptor signaling pathway; positive regulation of collateral sprouting; positive regulation of neuron projection development; positive regulation of non-membrane spanning protein tyrosine kinase activity; positive regulation of peptidyl-serine phosphorylation; positive regulation of receptor binding; positive regulation of synapse assembly; regulation of neuron differentiation; regulation of protein localization to cell surface; synapse assembly; transmembrane receptor protein tyrosine kinase signaling pathway
COX5A	Cytochrome c oxidase subunit 5A, mitochondrial	cytochrome-c oxidase activity; electron transfer activity; metal ion binding
COX6B1	Cytochrome c oxidase subunit 6B1	cytochrome-c oxidase activity
COX6C	Cytochrome c oxidase subunit 6C	cytochrome-c oxidase activity
COX7A2	Cytochrome c oxidase subunit 7A2, mitochondrial	cytochrome-c oxidase activity
COX7B	Cytochrome c oxidase subunit 7B, mitochondrial	cytochrome-c oxidase activity
COX7C	Cytochrome c oxidase subunit 7C, mitochondrial	cytochrome-c oxidase activity
COX8A	Cytochrome c oxidase subunit 8A, mitochondrial	cytochrome-c oxidase activity
NDUFA4	Cytochrome c oxidase subunit NDUFA4	NADH dehydrogenase (ubiquinone) activity; protein-containing complex binding; mitochondrial electron transport, cytochrome c to oxygen; mitochondrial electron transport, NADH to ubiquinone; proton transmembrane transport
NDUFA5	NADH dehydrogenase [ubiquinone] 1 alpha subcomplex subunit 5	NADH dehydrogenase (ubiquinone) activity; mitochondrial electron transport, NADH to ubiquinone; mitochondrial respiratory chain complex I assembly
NDUFB1	NADH dehydrogenase [ubiquinone] 1 beta subcomplex subunit 1	NADH dehydrogenase (ubiquinone) activity; mitochondrial electron transport, NADH to ubiquinone; mitochondrial respiratory chain complex I assembly
NDUFS4	NADH dehydrogenase [ubiquinone] iron-sulfur protein 4, mitochondrial	NADH dehydrogenase; brain development; cellular respiration; mitochondrial electron transport, NADH to ubiquinone; mitochondrial respiratory chain complex I assembly; positive regulation of fibroblast proliferation; reactive oxygen species metabolic process; regulation of protein phosphorylation; response to cAMP
POLR2E	DNA-directed RNA polymerases I, II, and III subunit RPABC1	a component of RNA polymerases I, II, and III which synthesizes ribosomal RNA precursors; catalyzes the transcription of DNA into RNA
PPARGC1A	Peroxisome proliferator-activated receptor gamma coactivator 1-alpha	alpha-tubulin binding; chromatin DNA binding; DNA binding; estrogen receptor binding; nuclear receptor binding; nuclear receptor coactivator activity; peroxisome proliferator-activated receptor binding; promoter-specific chromatin binding; RNA binding; sequence-specific DNA binding; transcription coactivator activity; transcription coregulator activity; transcription factor binding; ubiquitin protein ligase binding
SLC25A4	ADP/ATP translocase 1	adenine transmembrane transporter activity; ATP:ADP antiporter activity; oxidative phosphorylation uncoupler activity; proton transmembrane transporter activity; adaptive thermogenesis; ADP transport; apoptotic mitochondrial changes; generation of precursor metabolites and energy; mitochondrial ADP transmembrane transport; mitochondrial ATP transmembrane transport; mitochondrial genome maintenance; negative regulation of necroptotic process; positive regulation of mitophagy; regulation of mitochondrial membrane permeability; viral process
SLC25A5	ADP/ATP translocase 2	adenine nucleotide transmembrane transporter activity; adenine transmembrane transporter activity; ATP:ADP antiporter activity; oxidative phosphorylation uncoupler activity; proton transmembrane transporter activity; RNA binding; ubiquitin protein ligase binding; adaptive thermogenesis; adenine nucleotide transport; B cell differentiation; cellular response to leukemia inhibitory factor; chromosome segregation; erythrocyte differentiation; mitochondrial ADP transmembrane transport; mitochondrial ATP transmembrane transport; negative regulation of mitochondrial outer membrane permeabilization involved in apoptotic signaling pathway; positive regulation of cell population proliferation; positive regulation of mitophagy; regulation of mitochondrial membrane permeability
SP1	E3 ubiquitin-protein ligase SP1	bHLH transcription factor binding; cis-regulatory region sequence-specific DNA binding; DNA binding; DNA-binding transcription activator activity, RNA polymerase II-specific; DNA-binding transcription factor activity; DNA-binding transcription factor activity, RNA polymerase II-specific; double-stranded DNA binding; histone acetyltransferase binding; histone deacetylase binding; HMG box domain binding; metal ion binding; protein C-terminus binding; protein homodimerization activity; repressing transcription factor binding; RNA polymerase II cis-regulatory region sequence-specific DNA binding; RNA polymerase II repressing transcription factor binding; RNA polymerase II transcription regulatory region sequence-specific DNA binding; sequence-specific DNA binding; sequence-specific double-stranded DNA binding; transcription factor binding; transcription regulatory region sequence-specific DNA binding
TBP	TATA-box-binding protein	general transcription initiation factor activity; RNA polymerase II core promoter sequence-specific DNA binding; RNA polymerase II general transcription initiation factor activity; acrosome assembly; DNA-templated transcription, initiation; dTTP biosynthetic process; spermatid nucleus differentiation; transcription by RNA polymerase II
UQCRB	Cytochrome b-c1 complex subunit 7	aerobic respiration; mitochondrial electron transport, ubiquinol to cytochrome c; oxidative phosphorylation
UQCRH	Cytochrome b-c1 complex subunit 6, mitochondrial	ubiquinol-cytochrome-c reductase activity; aerobic respiration; mitochondrial electron transport, ubiquinol to cytochrome c; oxidative phosphorylation
VDAC1	Voltage-dependent anion-selective channel protein 1	ceramide binding; cholesterol binding; identical protein binding; ion channel binding; phosphatidylcholine binding; porin activity; protein-containing complex binding; protein kinase binding; voltage-gated anion channel activity

**Table 3:** Identified boar sperm zincoproteins of the HD pathway. Proteins were classified according to PANTHER classification system (http://pantherdb.org/) (accessed on 3 May 2021). Function(s) of the proteins were taken from the UniProtKB database (https://www.uniprot.org/), accessed on 3 May 2021.

Gene	Protein	Zinc-Binding or Containing?	Protein Class	Function(s)
ACTA1	Actin, alpha skeletal muscle	zinc-containing	actin and actin-related protein	ADP binding; ATP binding; myosin binding; structural constituent of cytoskeleton
ACTA2	Actin, aortic smooth muscle	zinc-containing	actin and actin-related protein	ATP binding; protein kinase binding
ACTB	Actin, cytoplasmic 1	zinc-containing	actin and actin-related protein	ATP binding; identical protein binding; kinesin binding; nitric-oxide synthase binding; protein kinase binding; structural constituent of cytoskeleton; structural constituent of postsynaptic actin cytoskeleton
ACTBL2	Beta-actin-like protein 2	zinc-containing	actin and actin-related protein	nucleosome binding; chromatin DNA binding; protein kinase binding; cell motility; ATP-dependent chromatin remodeling; axonogenesis
ACTC1	Actin, alpha cardiac muscle 1	zinc-containing	actin and actin-related protein	ATPase activity; ATP binding; myosin binding
ACTG1	Actin, cytoplasmic 2	zinc-containing	actin and actin-related protein	ATP binding; identical protein binding; profilin binding; structural constituent of cytoskeleton; structural constituent of postsynaptic actin cytoskeleton; ubiquitin protein ligase binding
ACTG2	Actin, gamma-enteric smooth muscle	zinc-containing	actin and actin-related protein	regulation of blood vessel diameter; smooth muscle contraction
ACTR2	Actin-related protein 2	zinc-containing	actin and actin-related protein	actin filament binding; actin filament polymerization; Arp2/3 complex-mediated actin nucleation
ARF1	ADP-ribosylation factor 1	zinc-containing	G-protein	anion binding; purine nucleotide binding; carbohydrate derivative binding; intracellular protein transport; vesicle-mediated transport
ARF2	ADP-ribosylation factor 4	zinc-containing	G-protein	anion binding; purine nucleotide binding; carbohydrate derivative binding; intracellular protein transport; vesicle-mediated transport
ARF3	ADP-ribosylation factor 3	zinc-containing	G-protein	anion binding; purine nucleotide binding; carbohydrate derivative binding; intracellular protein transport; vesicle-mediated transport
ARF6	ADP-ribosylation factor 6	zinc-containing	G-protein	anion binding; purine nucleotide binding; carbohydrate derivative binding; intracellular protein transport; vesicle-mediated transport
ARPC1A	Actin-related protein 2/3 complex subunit 1A	zinc-containing	actin or actin-binding cytoskeletal protein	actin filament binding; actin filament polymerization; Arp2/3 complex-mediated actin nucleation
CAPN1	Calpain-1 catalytic subunit	zinc-containing	cysteine protease	cysteine-type endopeptidase activity; proteolysis
CAPN11	Calpain-11	zinc-containing	cysteine protease	cysteine-type endopeptidase activity; proteolysis
CAPNS1	Calpain small subunit 1	zinc-containing	N/A	calcium-dependent cysteine-type endopeptidase activity; calcium ion binding
CLTA	Clathrin light chain A	zinc-containing	vesicle coat protein	clathrin binding; cellular protein-containing complex assembly; clathrin-dependent endocytosis; membrane invagination; vesicle budding from membrane
CLTB	Clathrin light chain B	zinc-containing	vesicle coat protein	clathrin binding; cellular protein-containing complex assembly; clathrin-dependent endocytosis; membrane invagination; vesicle budding from membrane
CRYZ	Quinone oxidoreductase	zinc-binding	oxidoreductase	identical protein binding; mRNA 3’-UTR binding; NADPH:quinone reductase activity; NADPH binding; zinc ion binding
CYC1	Cytochrome c1, heme protein, mitochondrial	zinc-binding	N/A	heme binding; metal ion binding; ubiquinol-cytochrome-c reductase activity
DCTN1	Dynactin subunit 1	zinc-containing	chaperone	microtubule binding; microtubule plus-end binding; protein kinase binding; tau protein binding; tubulin binding
DNAH1	Dynein heavy chain 1, axonemal	zinc-containing	microtubule-binding motor protein	ATPase activity, microtubule motor activity; protein binding; cilium movement
DNAH10	Dynein heavy chain 10, axonemal	zinc-containing	microtubule-binding motor protein	ATPase activity, microtubule motor activity; protein binding; microtubule-based movement
DNAH3	Dynein heavy chain 3, axonemal	zinc-containing	microtubule-binding motor protein	ATPase activity, microtubule motor activity; protein binding; cilium movement
DNAH5	Dynein heavy chain 5, axonemal	zinc-containing	microtubule-binding motor protein	ATPase activity, microtubule motor activity; protein binding; vesicle targeting; trans-Golgi to periciliary membrane compartment; ciliary transition zone assembly; protein localization to cilium; outer dynein arm assembly; intraciliary transport involved in cilium assembly
DNAH7 *	Dynein heavy chain 7, axonemal	zinc-containing	microtubule-binding motor protein	ATPase activity; microtubule motor activity; protein binding; cilium movement
DNAH8 *	Dynein heavy chain 8, axonemal	zinc-containing	microtubule-binding motor protein	ATPase activity, microtubule motor activity; protein binding; vesicle targeting; trans-Golgi to periciliary membrane compartment; ciliary transition zone assembly; protein localization to cilium; outer dynein arm assembly; intraciliary transport involved in cilium assembly
DNAI2 *	Dynein intermediate chain 2, axonemal	zinc-containing	microtubule or microtubule-binding cytoskeletal protein	dynein heavy chain binding; vesicle targeting; trans-Golgi to periciliary membrane compartment; ciliary transition zone assembly; cilium movement; protein localization to cilium; outer dynein arm assembly; intraciliary transport involved in cilium assembly
DNAL4	Dynein light chain 4, axonemal	zinc-containing	microtubule or microtubule-binding cytoskeletal protein	protein binding; positive regulation of hydrolase activity
DYNC1H1	Cytoplasmic dynein 1 heavy chain 1	zinc-containing	microtubule-binding motor protein	ATPase activity; microtubule motor activity; protein binding; vesicle transport along microtubule; mitotic nuclear division; establishment of spindle localization; cytoplasmic microtubule organization; nuclear migration
DYNC1I2	Cytoplasmic dynein 1 intermediate chain 2	zinc-containing	microtubule or microtubule-binding cytoskeletal protein	dynein heavy chain binding; transport along microtubule
DYNLL2	Dynein light chain 2, cytoplasmic	zinc-containing	microtubule or microtubule-binding cytoskeletal protein	protein binding; vesicle targeting, trans-Golgi to periciliary membrane compartment; ciliary transition zone assembly; positive regulation of hydrolase activity; protein localization to cilium; intraciliary transport involved in cilium assembly; axoneme assembly
GAPDH	Glyceraldehyde-3-phosphate dehydrogenase	zinc-containing	dehydrogenase	oxidoreductase activity; oxidation-reduction process; glycolytic process
GAPDHS	Glyceraldehyde-3-phosphate dehydrogenase, testes-specific	zinc-containing	dehydrogenase	oxidoreductase activity; oxidation-reduction process; glycolytic process
HIP1 *	Huntingtin-interacting protein 1	zinc-containing	non-motor actin-binding protein	phosphatidylinositol bisphosphate binding; protein-macromolecule adaptor activity; actin filament binding; clathrin binding; positive regulation of cellular component organization; actin filament organization; regulation of receptor-mediated endocytosis; apoptotic process; cellular protein metabolic process; cellular protein-containing complex assembly; positive regulation of transport receptor-mediated endocytosis; proteolysis; membrane invagination; activation of cysteine-type endopeptidase activity involved in apoptotic process vesicle budding from membrane
HIP1R	Huntingtin-interacting protein 1-related protein	zinc-containing	non-motor actin-binding protein	phosphatidylinositol bisphosphate binding; protein-macromolecule adaptor activity; actin filament binding; clathrin binding; positive regulation of cellular component organization; actin filament organization; regulation of receptor-mediated endocytosis; apoptotic process; cellular protein metabolic process; cellular protein-containing complex assembly; positive regulation of transport receptor-mediated endocytosis; proteolysis; membrane invagination; activation of cysteine-type endopeptidase activity involved in apoptotic process vesicle budding from membrane
RAB8A	Ras-related protein Rab-8A	zinc-containing	N/A	regulation of exocytosis; vesicle fusion to plasma membrane; protein secretion; protein localization to plasma membrane; organelle localization; neurotransmitter receptor transport to postsynaptic membrane; cellular response to insulin stimulus; Golgi vesicle transport
TUBB	Tubulin beta chain	zinc-containing	tubulin	structural molecule activity; anion binding; purine nucleotide binding; carbohydrate derivative binding; microtubule cytoskeleton organization; mitotic nuclear division
TUBB4A	Tubulin beta-4A chain	zinc-containing	tubulin	structural molecule activity; anion binding; purine nucleotide binding; carbohydrate derivative binding; microtubule cytoskeleton organization; mitotic nuclear division
TUBB4B *	Tubulin beta-4B chain	zinc-containing	tubulin	structural molecule activity; anion binding; purine nucleotide binding; carbohydrate derivative binding; microtubule cytoskeleton organization; mitotic nuclear division
TUBB6	Tubulin beta-6 chain	zinc-containing	tubulin	structural molecule activity; anion binding; purine nucleotide binding; carbohydrate derivative binding; microtubule cytoskeleton organization; mitotic nuclear division
VAT1	Synaptic vesicle membrane protein VAT-1 homolog	zinc-binding	N/A	oxidoreductase activity; zinc ion binding

* Significantly different proteins in abundance after in vitro capacitation.

## Data Availability

No new data were created or analyzed in this study.
